# PLA-Based Mineral-Doped Scaffolds Seeded with Human Periapical Cyst-Derived MSCs: A Promising Tool for Regenerative Healing in Dentistry

**DOI:** 10.3390/ma12040597

**Published:** 2019-02-16

**Authors:** Marco Tatullo, Gianrico Spagnuolo, Bruna Codispoti, Fausto Zamparini, Anqi Zhang, Micaela Degli Esposti, Conrado Aparicio, Carlo Rengo, Manuel Nuzzolese, Lucia Manzoli, Fabio Fava, Carlo Prati, Paola Fabbri, Maria Giovanna Gandolfi

**Affiliations:** 1 Tecnologica Research Institute, Stem Cell Unit, 88900 Crotone, Italy; marco.tatullo@tecnologicasrl.com (M.T.); bruna.codispoti@tecnologicasrl.com (B.C.); 2 Department of Neurosciences, Reproductive and Odontostomatological Sciences, University of Naples “Federico II”, 80131 Napoli, Italy; gianrico.spagnuolo@gmail.com; 3 Laboratory of Biomaterials and Oral Pathology, School of Dentistry, Department of Biomedical and Neuromotor Sciences, University of Bologna, 40125 Bologna, Italy; fausto.zamparini2@unibo.it (F.Z.); carlo.prati@unibo.it (C.P.); 4 MDRCBB-Minnesota Dental Research Center for Biomaterials and Biomechanics, School of Dentistry, University of Minnesota, Minneapolis, MN 55455, USA; azhang@umn.edu (A.Z.); apari003@umn.edu (C.A.); 5 Department of Civil, Chemical, Environmental and Materials Engineering, University of Bologna, 40131 Bologna, Italy; micaela.degliesposti@unibo.it (M.D.E.); fabio.fava@unibo.it (F.F.); p.fabbri@unibo.it (P.F.); 6 Department of Prosthodontics and Dental Materials, School of Dental Medicine, University of Siena, 53100 Siena, Italy; carlo.rengo@alice.it; 7 University Hospitals Birmingham—NHS Foundation Trust, Birmingham B152GW, UK; manuelnuzzolese@libero.it; 8 Cellular Signalling Laboratory, Institute of Human Anatomy, Department of Biomedical and Neuromotor Sciences, University of Bologna, 40126 Bologna, Italy; lucia.manzoli@unibo.it

**Keywords:** polylactic acid (PLA)-based scaffolds, calcium silicate-containing scaffolds, bioactive scaffolds, porous scaffolds, human periapical cyst mesenchymal stem cells (hPCy-MSCs), regenerative dentistry, thermally induced phase separation technique (TIPS)

## Abstract

Human periapical cyst mesenchymal stem cells (hPCy-MSCs) are a newly discovered cell population innovatively collected from inflammatory periapical cysts. The use of this biological waste guarantees a source of stem cells without any impact on the surrounding healthy tissues, presenting a valuable potential in tissue engineering and regenerative medicine applications. In the present study, hPCy-MSCs were collected, isolated, and seeded on three experimental mineral-doped porous scaffolds produced by the thermally-induced phase-separation (TIPS) technique. Mineral-doped scaffolds, composed of polylactic acid (PLA), dicalcium phosphate dihydrate (DCPD), and/or hydraulic calcium silicate (CaSi), were produced by TIPS (PLA-10CaSi, PLA-5CaSi-5DCPD, PLA-10CaSi-10DCPD). Micro-CT analysis evaluated scaffolds micromorphology. Collected hPCy-MSCs, characterized by cytofluorimetry, were seeded on the scaffolds and tested for cell proliferation, cells viability, and gene expression for osteogenic and odontogenic differentiation (DMP-1, OSC, RUNX-2, HPRT). Micro-CT revealed an interconnected highly porous structure for all the scaffolds, similar total porosity with 99% open pores. Pore wall thickness increased with the percentage of CaSi and DCPD. Cells seeded on mineral-doped scaffolds showed a superior proliferation compared to pure PLA scaffolds (control), particularly on PLA-10CaSi-10DCPD at day 12. A higher number of non-viable (red stained) cells was observable on PLA scaffolds at days 14 and 21. DMP-1 expression increased in hPCy-MSCs cultured on all mineral-doped scaffolds, in particular on PLA-5CaSi-5DCPD and PLA-10CaSi-10DCPD. In conclusion, the innovative combination of experimental scaffolds colonized with autologous stem cells from periapical cyst represent a promising strategy for regenerative healing of periapical and alveolar bone.

## 1. Introduction

The main stem cell ability to regenerate injured tissues confers them a central role in regenerative medicine. The first success obtained with hematopoietic stem cells encouraged studies on stem cell therapy for the treatment of several human diseases [1,2].

Mesenchymal stem cells (MSCs) retain both the proliferation ability and the capability to modulate inflammatory and immune response. These peculiar features strongly triggered the searching for novel sources of MSCs. The early MSCs were successfully isolated from bone marrow (hBM): surgical harvesting of hBM-MSCs is not a simple procedure, in fact, patients must undergo an invasive surgery to aspirate bone marrow from the iliac crests, and cells obtained from hBM are typically few [3]. The numerous issues related to MSCs collection have attracted the interest of researchers in discovering alternative sources of MSCs, in order to obtain them without any invasive or painful procedure.

Dental pulp infections and necrosis could lead to the formation of inflammatory tissues, richly infiltrated by macrophages, neutrophils, and lymphocytes, in the periapical region (periapical granuloma), with a consequent onset of apical periodontitis. The chronicization of such an inflammatory condition may evolve towards periapical cyst formation [4]. Endodontic inflammatory cysts have a high prevalence and they increase in elderly patients: 5% of teeth and 25% of teeth treated endodontically can have a periapical x-ray discovered lesion [5]. These data are probably highly under-estimated [6]. 

Recently, human periapical cyst-derived mesenchymal stem cells (hPCy-MSCs) were isolated by Italian researchers [7]. hPCy-MSCs population share the same phenotype with other types of oral derived MSCs, such as dental pulp stem cells. Such cells are characterized by high expression of stemness-related markers: CD13, CD29, CD44, CD73, CD90, CD105, and some hematopoietic markers, such as CD45 and CD34. [8,9] The high expression of CD146 on the surface of hPCy-MSCs is also linked to the multipotency of these cells [10]. hPCy-MSCs showed a great plasticity, showing a surprising odontogenic/osteogenic [11] and a neurogenic commitment [12] under specific conditions. The epithelial wall of inflammatory cysts represents an attractive source of mesenchymal stem cells to be used in regenerative medicine with special regards to orthopedic and dental applications [3]. Oral-derived stem cells associated with specific biomaterials are currently used in many tissue engineering procedures [13], with special regards to bone regeneration [14,15]. However, the right scaffold is still a challenging searching, in order to merge the best MSCs with the best scaffold.

When considering tissue engineering, a 3D porous scaffold is necessary for cells attachment, proliferation, differentiation, new blood vessels ingrowth, and, finally, new tissue formation [16]. An ideal scaffold for bone application should be extremely porous, biocompatible, biointeractive (able to release biologically relevant ions), with tailored resorption and adequate mechanical properties. Combining a polymeric matrix with one or more bioactive or biointeractive filler represents an interesting strategy in bone tissue engineering. Composite materials usually show an excellent balance between the strengths and weaknesses of their individual components. In this way, the fabrication of polymeric scaffolds is much easier and less time-consuming compared to ceramic scaffolds, which are also brittle and less versatile [17]. Calcium phosphate compounds such as hydroxyapatite and dicalcium and tricalcium phosphates are some of the most extensively fillers used in bone tissue engineering [18]. These materials alone however, cannot provide the necessary biointeractive, bioactive, and mechanical properties for dental tissue engineering. Hydroxyapatite doped scaffolds demonstrated osteoconductive and long-term bone tissue stability, but revealed significant brittleness at high loadings [18,19], while b-TCP is highly-interactive but fast-resorbing and unable to provide a long-term template to support cells migration from the periphery of the grafted area [20,21].

Calcium silicates (CaSi) are hydraulic cements widely used in regenerative endodontics in close vicinity to pulp or bone tissue [22,23,24]. Nowadays, CaSi-based materials are used in several endodontic applications, such as root canal sealing [25,26], root-end filling materials [27], and for direct pulp capping [22]. CaSi biointeractivity and the property to nucleate calcium phosphates/apatite suggest their pivotal role in mineral tissue regeneration by activating the osteogenic potential and promoting the differentiation of mineralizing-cells [24]. The combination of reactive calcium phosphates (such as dicalcium phosphate dehydrate, DCPD) to CaSi materials demonstrated to enhance their biological properties and apatite-forming ability [28,29].

Several techniques have been developed to produce polymeric scaffolds for tissue engineering, including electrospinning technique [30], 3D printing [31], salt leaching [32], and thermally induced phase separation (TIPS) [33,34]. Hybrid Poly-e-caprolactone-polyglycolic acid solution was used to produce biodegradable scaffolds by a 3D mold for human tooth-ligament in dental applications [35]. Then, polymeric composites containing biphasic calcium phosphate (80:20 wt %) prepared by hot melt extrusion (100 °C) for 3D printing were able to support the differentiation of human dental pulp stem cells into osteogenic lineage in calvarial defects [36]. Polymeric composites doped with 10–50 wt % of β-tricalcium phosphate produced by a foaming agent and heating at 90 °C demonstrated to promote proliferation of human bone marrow mesenchymal stem cells also supporting the differentiation to reparative hard tissue [37]. All these materials, however, failed to comply with porosity values over 90%.

TIPS is an experimental procedure to produce highly porous scaffolds without using high temperatures (which may alter the fillers properties). Our recent study found that a Polylactic Acid (PLA) polymeric scaffold doped with considerable amounts of bioactive and biointeractive fillers (up to 20%) and having a bulk porosity higher than 90% may be produced by TIPS [34].

The association of these promising scaffolds with hPCy-MSCs may result as an interesting regenerative approach in periapical regeneration and healing, where scaffolds with biointeractive bone-stimulating properties may be placed in the presence of large periapical bone defects.

Therefore, the aim of this study was to test the biological activity of mineral-doped CaSi-DCPD porous scaffolds by human periapical cyst-mesenchymal stem cells (hPCy-MSCs). 

## 2. Materials and Methods

### 2.1. Materials

Poly(L-lactic acid MW = 65,000 g/mol) (IngeoTM biopolymer PLA 4060D, Natureworks LLC, Blair, NE, USA) was used. Methanol (MeOH), ethanol (EtOH, 99.8%), 1,4-dioxan (DIOX), and chloroform (CHCl_3_, HPLC grade) all from Sigma Aldrich (Milan, Italy) were used as received without further purification. PLA was received in pellet form and purified via dissolution in CHCl_3_ (10% wt/vol) and reprecipitation in a large excess of cold MeOH, in order to eliminate residual polymerization catalysts [33]. Dicalcium phosphate dihydrate (DCPD; CaHPO_4_·2H_2_O) powders (Sigma-Aldrich, Steinheim, Germany) [22,29] and/or calcium silicate (CaSi) powders (Aalborg, Denmark) [23] composed of dicalcium silicate, tricalcium silicate, tricalcium aluminate, and calcium sulfate were prepared by melt-quenching technique and milling procedures [34].

### 2.2. TIPS Scaffolds Preparation

Porous mineral-doped scaffolds were prepared starting from PLA solutions in DIOX (3.5% wt/vol) as solvent and porogen agent; CaSi and DCPD powders were added to the PLA solution in amounts 5% or 10% by weight with respect to PLA. Homogeneous dispersions were obtained by sonicating the mixtures for 3 h using an ultrasonic processor (UP50H, Hielsher Ultrasonics, Teltow, Germany) working at 50 watts and 30 kHz, equipped with a sonotrode MS2 (made of titanium, tip diameter 2 mm). After mixing, solutions were placed inside disposable aluminium dishes of 60 mm in diameter, and cooled at −18 °C. After 18 h, the frozen samples were removed from the dishes and fully immersed for 48 h in EtOH bath precooled at −18 °C, with solvent refresh every 3 h for the DIOX extraction procedure. Then the porous samples were placed under vacuum and completely dried [33,34].

The prepared scaffolds were: PLA-10CaSi, PLA-5CaSi-5DCPD, PLA-10CaSi-10DCPD, and PLA as control.

### 2.3. Scaffold Characterization by Micro-CT

**Figure 1:**
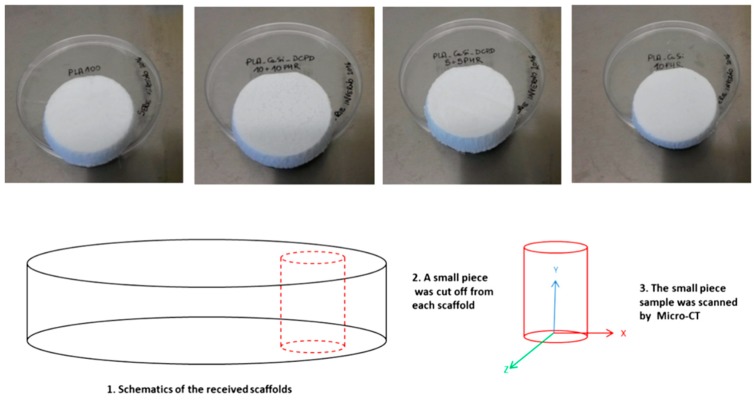
Highly-porous polylactic acid (PLA) scaffolds added with dicalcium phosphate dihydrate (DCPD) and/or hydraulic calcium silicate (CaSi) used in this study: Micro-CT scanning slices protocol.

A coring/cylindrical section of each scaffold formulation has been used for micro-CT analysis (HMX-XT 225 X-tek system, Nikon, Minneapolis, MI, USA) (Figure 1). The scanning parameters used were 80 KV voltage, 69 µA Current, 35.3 magnification, 354 ms exposure time length, and 30,000 Shading correction white Target. The resolution of the scanned images was 5.66 μm. Two randomly selected cross-sectional slices of each scanned scaffold sample were shown here. All the images were adjusted to the same grey value range.

The total porosity, open porosity, and the average wall thickness of each sample were analyzed based on the reconstructed 3D structure. After scanning, reconstruction of the original scanning pictures was performed using CT Pro 3D XT 3.1.11 (Nikon Metrology Brighton, MI, USA). VGSTUDIO MAX 3.1 (Volume Graphics Charlotte, NC, USA) was used to export slices of the reconstructed images to .*dicom* format, so that porosity and wall thickness analysis of the scaffolds could be quantified using CT Analyser 1.14.4.1+ (2012-14 Bruker microCT). For the porosity and wall thickness analysis, 16-bit .*dicom* slices were transferred to binary images. A proper threshold range was selected to show the scaffold structure in the binary version. After that, the binary images were transferred into the *Morphometry* version. The comparison between 16-bit .*dicom* version and the transferred *Morphometry* version was done for multiple slices of each sample to guarantee the selected threshold was reliable enough to show the scaffold structure as much as possible. With the images determined for optimal *Morphometry* the 3D total porosity, the 3D open porosity, and the average wall thickness were calculated for the whole 3D structure.

### 2.4. Cell Culture

Human third molars affected by massive caries were obtained after written informed consent from volunteers and collaborators requiring teeth extraction for severe pulp necrosis and local inflammation; after tooth extraction, inflammatory periapical cystic tissues were removed along with necrotic tooth. Surgical procedures were performed at Calabrodental Dental clinic in Crotone, Italy (ethical committee agreement code was: CBD-021/TRI/2016). All clinical investigations have been conducted according to the principles reported in the Declaration of Helsinki.

The isolation of human periapical cyst-derived mesenchymal stem cells (hPCy-MSCs) was obtained by the enzymatic digestion of cystic wall, with the aim to collect MSCs to further characterize in the following steps.

More in details, the cystic tissue was washed 5 times with phosphatase buffer saline (PBS, Corning, Manassas, VA, USA) added with 1% penicillin-streptomycin antibiotics (Invitrogen, 15140122, Carlsbad, CA, USA), and 2.5 μg/mL amphotericin B antimycotic (Invitrogen, 15290026, Carlsbad, CA, USA).

Then, the tissue was minced with a sterile scalpel and placed into a PBS solution, containing 3 mg/mL type I collagenase (Invitrogen, 17100-017, Carlsbad, CA, USA) with 4 mg/ml dispase (Sigma, D4818, Milan, Italy) for 2 h at 37 °C for a proper enzymatic digestion. The obtained solution was filtered, and the cells were collected after centrifugation at 1500RPM for 10 min: cells were plated in alpha-minimal essential medium (α-MEM) (Invitrogen, Carlsbad, CA, USA) added with 10% foetal bovine serum (FBS; Invitrogen, Carlsbad, CA, USA), 2mM glutamine, P/S (Invitrogen Carlsbad, CA, USA), and amphotericin-B (Invitrogen Carlsbad, CA, USA). 

These cells were finally incubated at 37 °C and 5% CO_2_, and the medium was replaced bi-weekly. Different batches of cells were used for this specific study.

### 2.5. Cytofluorimetric Analysis

Isolated cells were phenotypically investigated for the expression of mesenchymal stem cell-like markers using the following antibodies: anti-CD13 (PE, 560998, Becton Dickinson, San Jose, CA, USA), anti-CD90 (PE, 555596, Becton Dickinson, San Jose, CA, USA), anti-CD105 (APC, 562408, Becton Dickinson, San Jose, CA, USA), anti-CD73 (FITC, 561254, Becton Dickinson, San Jose, CA, USA), anti-CD146 (PE, sc-18837, Santa Cruz Biotechnology, Inc), anti-CD44 (FITC, 560977, Becton Dickinson, San Jose, CA, USA), and anti-CD29 (APC, 561794, Becton Dickinson, San Jose, CA, USA).

The absence of hematopoietic markers was assessed using anti-CD34 (FITC, 130-098-142, Miltenyi Biotec, Bergisch Gladbach, Germania), anti-CD45 (APC-H7, 560178, Becton Dickinson, EU), and anti-HLA-DR (PE, 130-104-873, Miltenyi Biotec, Bergisch Gladbach, Germania) antibodies. Cytofluorimetric measurements were performed using a NAVIOS instrument (Navios Flow Cytometer, Beckman Coulter, Life Sciences, Indianapolis, IN, USA) and the Kaluza 1.3 program (Kaluza Analysis Software, Beckman Coulter, Life Sciences, Indianapolis, IN, USA) was used for data analysis.

### 2.6. Proliferation Assay

The scaffolds were sized as 1 cm^2^ squared pieces, with a sterile scalpel (0.5 cm thickness), and sterilized by UV-rays exposure for 2 h, and finally pre-incubated for 2 h with complete medium in 24-wells plates.

HPCy-MSCs were seeded in concentration of 7 × 10^5^ on PLA-10CaSi, PLA-5CaSi-5DCPD and PLA-10CaSi-10DCPD scaffolds and incubated in α-MEM complete medium (see above) at 37 °C and 5% CO_2_ enriched atmosphere; pure PLA scaffolds were used as control. 

The same number of cells were plated on tissue culture treated 24-well plate as growth control. 

Presto Blue reagent (Invitrogen, A13261, Carlsbad, CA, USA) has been used as metabolic assay for the evaluation of cell growth. Presto Blue reagent was diluted according to manufacturer’s instructions and added to the wells containing the scaffolds seeded with hPCy-MSCs or the empty scaffolds (negative control).

After incubation for 2 h at 37 °C and 5% CO_2_, the absorbance of resazurin dye was measured using a Multiskan GO (Thermo Fisher N10588, Waltham, MA, USA) spectrophotometer at 570–600 nm wavelength. Metabolic assays were performed at days 3, 7, 10, and 14.

### 2.7. Live/Dead Assay

The presence of viable and/or non-viable cells seeded on scaffolds was detected by staining samples with a solution of 2 μM Calcein AM (acetoxymethyl) and 4 μM EthDIII (Ethidium Homodimer III, Biotium, Hayward, 30002, CA, USA) in PBS. Calcein AM stains viable cells by emitting a green fluorescent signal (FITC) instead EthD-III stains only dead cells in red (rhodamine). 

After 2 washings with PBS, samples were incubated with the staining-solution for 40 min at room temperature.

Fluorescent signal was revealed through a confocal laser scanning microscope (CLSM) (SP5, Leica, Wetzlar, Germany) and relative images were acquired by a microscope associated camera.

Live/dead assay has been performed at days 7, 14, and 21.

### 2.8. qPCR

After 21 days of cell culture, scaffolds seeded with hPCy-MSCs were washed with PBS and then cells have been harvested by using TRIzol reagent (Thermo Fisher, Carlsbad, CA, USA).

The Purelink™ RNA mini kit (Applied Biosystems, Vilnius, Lithuania) was used for RNA extraction, quantification of total RNA was performed by using a Multiskan Go spectrophotometer (Thermo scientific, Waltham, MA, USA). Total RNA samples (250 ng) were subjected to the reverse-transcription reaction using the High Capacity RNA-to-cDNA Kit (Applied Biosystems, Vilnius, Lithuania). cDNA samples were amplified by real-time PCR with the power SYBR green PCR Master Mix (Applied Biosystems, Vilnius, Lithuania) with 2 pmol of primers in a total volume of 10 µL.

Real-time PCR reactions were performed using a Pikoreal 96 (Thermo Fisher, Carlsbad, CA, USA) apparatus with the following conditions: initial denaturation step at 95 °C for 10 min, followed by 40 cycles of 10 s at 95 °C and 1 min at 60 °C. Relative expression levels were calculated using the ΔΔCt method after normalization to the expression of the HRPT housekeeping gene. 

Primer sequences for Runt-related transcription factor-2 (RUNX-2), dentin matrix protein-1 (DMP-1) and osteocalcin (OSC) genes are listed below:

RUNX-2
For: ATGTGTGTTTGTTTCAGCAGCA
Rev: TCCCTAAAGTCACTCGGTATGTGTA
DMP-1
For: GTGAGTGAGTCCAGGGGAGATAA
Rev: TTTTGAGTGGGAGAGTGTGTGC
OSC
For: TGAGAGCCCTCACACTCCTC
Rev: ACCTTTGCTGGACTCTGCAC

### 2.9. Statistical Analysis

Results are expressed as the mean ± standard deviation (SD) of three specific different experiments.

## 3. Results

### 3.1. Scaffolds Characterization by Micro-CT

**Figure 2:**
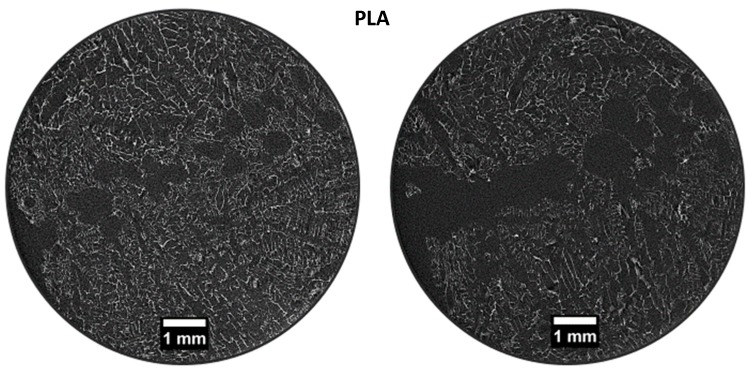
Micro-CT scan of 2 random slices of PLA scaffold, revealing a heterogeneous highly porous structure with a central empty area, attributable to sample brittleness.

**Figure 3:**
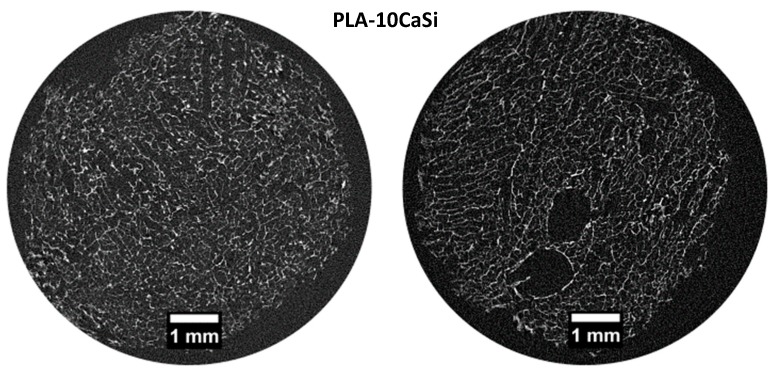
Micro-CT scan of 2 random slices of PLA-10CaSi shows highly porous, more regular “honey-comb” like structure. Small CaSi granules with higher radiopacity may be identified on all the scaffold structure.

In the PLA scaffold (control), micro-CT scan of 2 random slices revealed a heterogeneous highly porous structure (Figure 2). Pores appeared not to be uniform in size and shape with macro-pores ranging from 0.5 to 1.5 mm and large empty spaces in the central part of the structure. PLA-10CaSi scaffolds had a highly porous, “honey-comb” like, and more regular structure (Figure 3) than the control scaffolds. Differently from the pure PLA sample, no empty areas were present in the central portion of the PLA-10CaSi scaffold. However, large pores (approx. 1 mm in radius) were evident in some parts of the scaffold. Smaller regular-shaped pores constituted most of the structure of these scaffolds. The small granules of the minerals conferred a higher radiopacity to these scaffolds compared to the control scaffolds, which allowed visualization of a more defined structure and indicated the mineral particles were well-distributed along the whole scaffold volume. 

**Figure 4:**
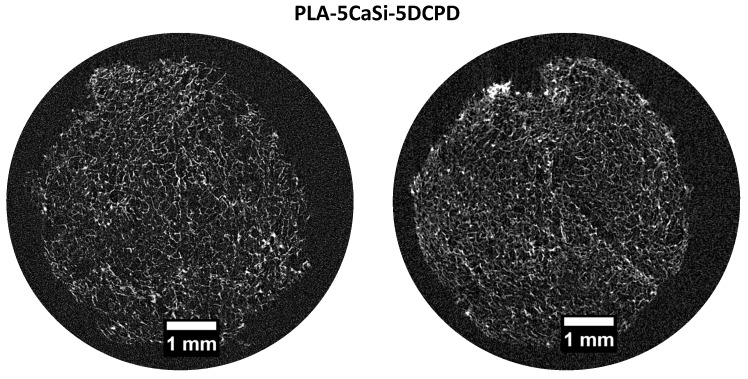
Micro-CT scan of 2 random slices of PLA-5CaSi-5DCPD reveals a highly porous regular “honey-comb” like structure, similar to the other mineral doped scaffolds. Some pores are occluded by radiopaque granules.

**Figure 5:**
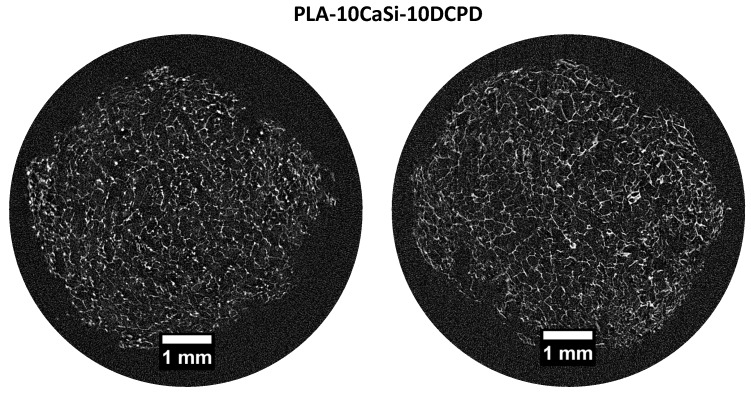
Micro-CT scan of 2 random slices of PLA-10CaSi-10DCPD reveals a highly porous regular “honey-comb like structure”, similar to the other mineral-doped scaffolds.

**Figure 6:**
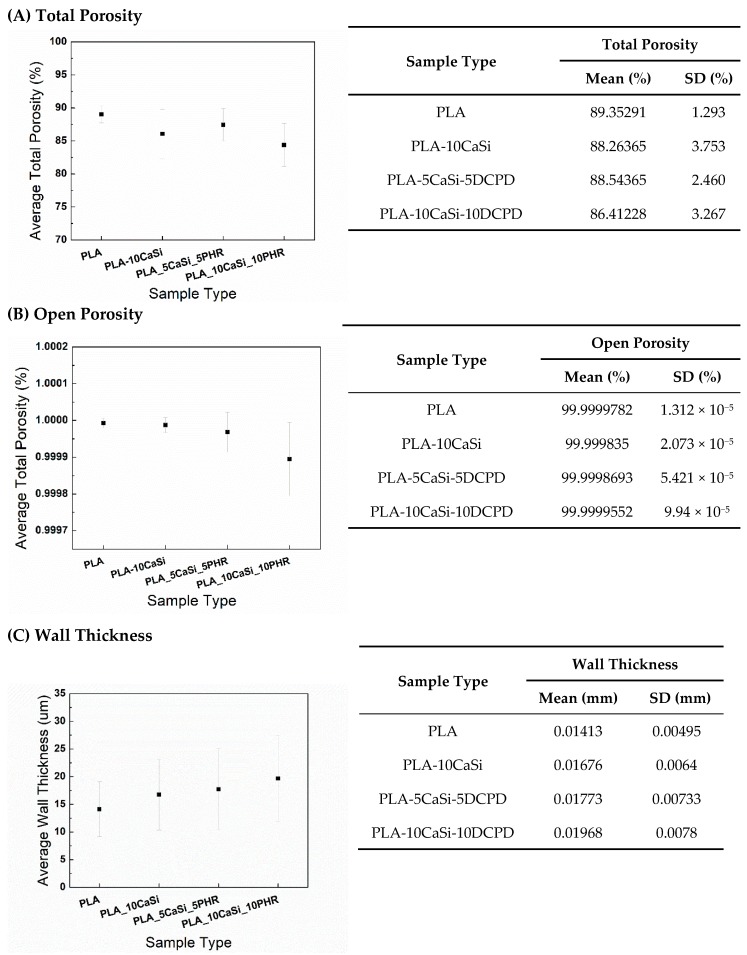
Quantification of % of total porosity (**A**), % of open porosity (**B**), and average wall thickness of the pores (**C**) from the micro-CT scanned samples of all different scaffolds.

PLA-5CaSi-5DCPD (Figure 4) and scaffold PLA-10CaSi-10DCPD (Figure 5) had very similar structures to the one of the PLA-10CaSi scaffold, including highly porous, regular “honey-comb” like structure with homogenous small pores and well distributed radiopaque mineral granules. However, at the edge of the scaffolds a higher concentration of CaSi and DCPD fillers can be identified. All tested scaffolds had a high, similar percentage of total porosity (86.4–89.4%) and open porosity (>99%), but the wall thickness of the pores increased with the amount of minerals used to load the scaffold (Figure 6). The open pore structure is an important feature for promoting colonization and degradation of the scaffolds. Also, the low standard deviation in the thickness of the pore walls corroborates the homogeneous distribution of the minerals in the structure of all the scaffolds.

### 3.2. Biological Assays

**Figure 7:**
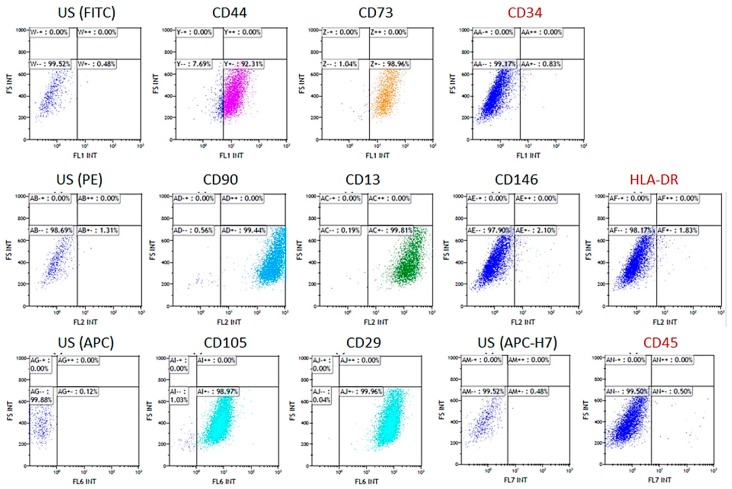
Cytofluorimetric analysis. Phenotypic expression pattern of primary cells isolated from human periapical cyst.

The cellular response of the recently described human periapical cyst-mesenchymal stem cells, seeded on highly-porous PLA-based scaffolds, mineral-doped with dicalcium phosphate dihydrate and/or hydraulic calcium silicate, has been investigated. Impressively high expression of mesenchymal stem cell-like markers was detected; in particular: CD90, CD105, CD73, CD44, CD29, and CD13 were highly expressed by cells isolated from human periapical cysts, so demonstrating their immature phenotype and the great regenerative potential of this MSC population meritoriously isolated from wasted biological tissues. The multipotent mesenchymal stromal nature of hPCy-MSCs was further confirmed by the lack of the expression of hematopoietic surface markers CD34, CD45, and HLA-DR (Figure 7). 

### 3.3. Metabolic Assay

The proliferation rate of hPCy-MSCs has been quantified by using a resazurin-based metabolic assay (Presto Blue Invitrogen, A13261). The metabolic activity of live cells allows the conversion of resazurin into an optically-detectable component; the rate of resazurin conversion directly correlates with the number of viable cells present in the culture.

**Figure 8:**
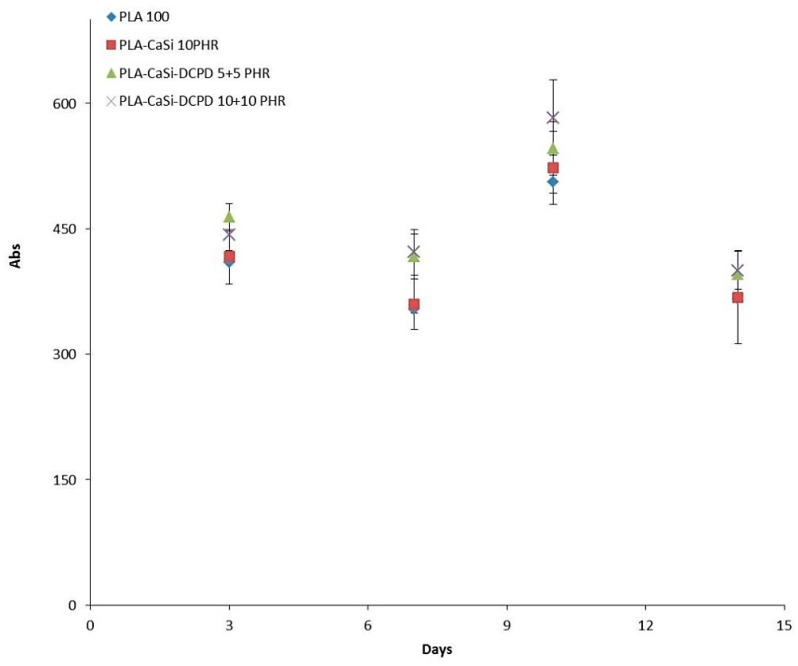
Proliferation curve. Quantitative results of hPCy-MSCs proliferation seeded on PLA scaffolds measured by the Prestoblue metabolic activity assay (absorbance (Abs) in the Y axis) at days 3, 7, 10, and 14 of culture (Time in the X axis). Error bars represent standard deviations.

Cell proliferation was monitored at different time-points (3, 7, 10, and 14 days, after the seeding). Figure 8 shows the same hPCy-MSCs’ growing trend, in all the experimental samples, with a maximum peak reached at day 10, followed by a slightly decline at day 14. Interestingly, cells seeded on mineral-doped scaffolds showed a better proliferation ability, compared to the PLA-control group; this behavior was confirmed for those cells seeded on PLA-10CaSi-10DCPD scaffolds, particularly at day 10.

### 3.4. Live/Dead Assay

The presence of live and dead cells after seeding on the experimental scaffolds at 7, 14, and 21 days of culture was examined by confocal analysis, by using specific Calcein AM-EthDIII live/dead fluorescent staining.

This assay provides green/red fluorescent staining of viable and non-viable cells, respectively; briefly, Calcein AM penetrates into live cells and is cleaved by the cytoplasmic esterases to yield the green fluorescent dye and is retained thought membrane integrity of viable cells. EthD-III enters only dead cells with compromised plasma membranes and binds DNA, thus marks the nucleus with bright red fluorescence.

**Figure 9:**
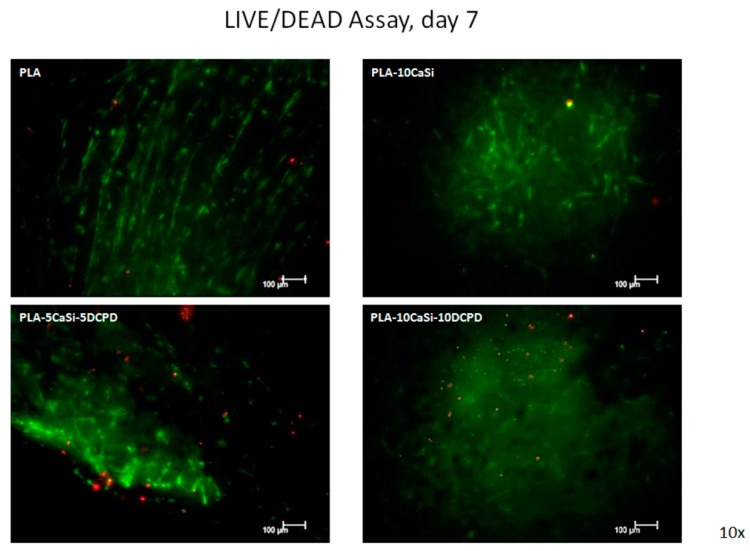
Live/dead assay. Live/dead staining, at day 7 of culture, in order to detect viable (green) and non-viable (red) hPCy-MSCs within the tested scaffolds (CLSM, 10× magnification).

**Figure 10:**
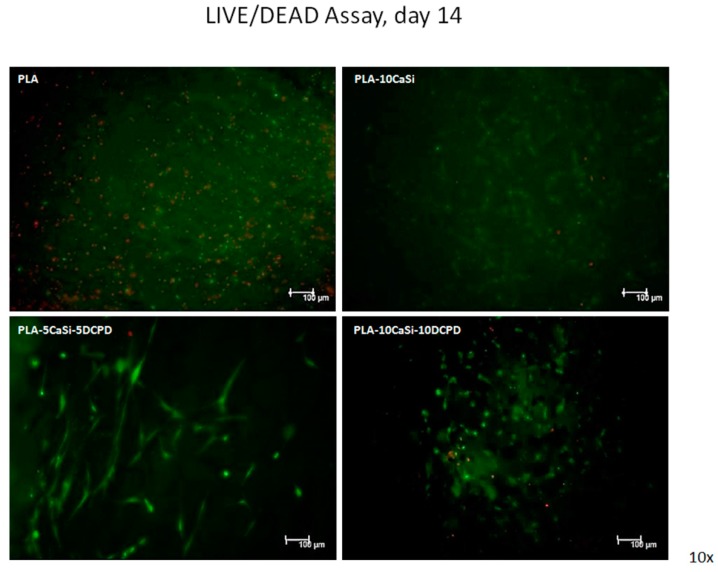
Live/dead assay. Live/dead staining, at day 14 of culture, in order to detect viable (green) and non-viable (red) hPCy-MSCs within the tested scaffolds (CLSM, 10× magnification).

**Figure 11:**
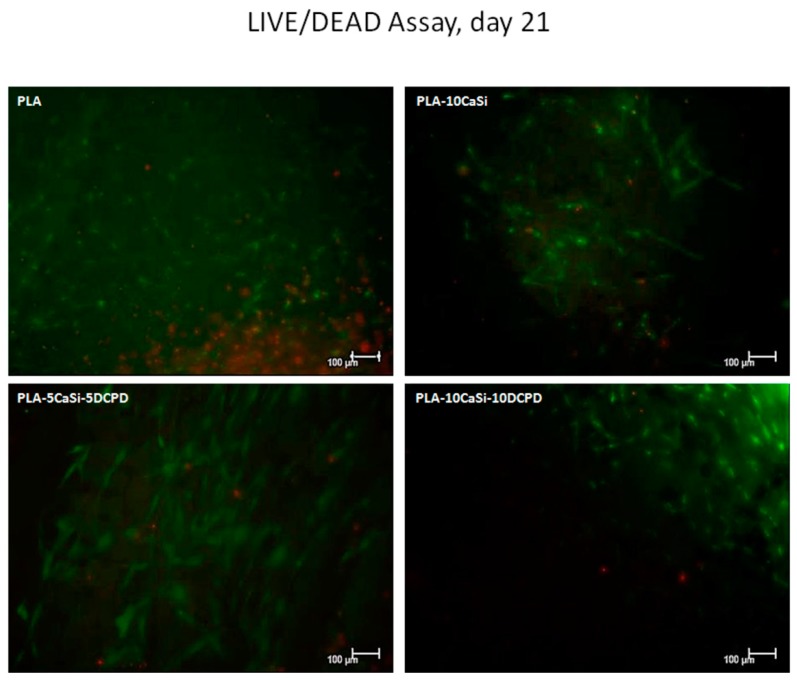
Live/dead assay. Live/dead staining, at day 21 of culture, in order to detect viable (green) and non-viable (red) hPCy-MSCs within the tested scaffolds (CLSM, 10× magnification).

**Figure 12:**
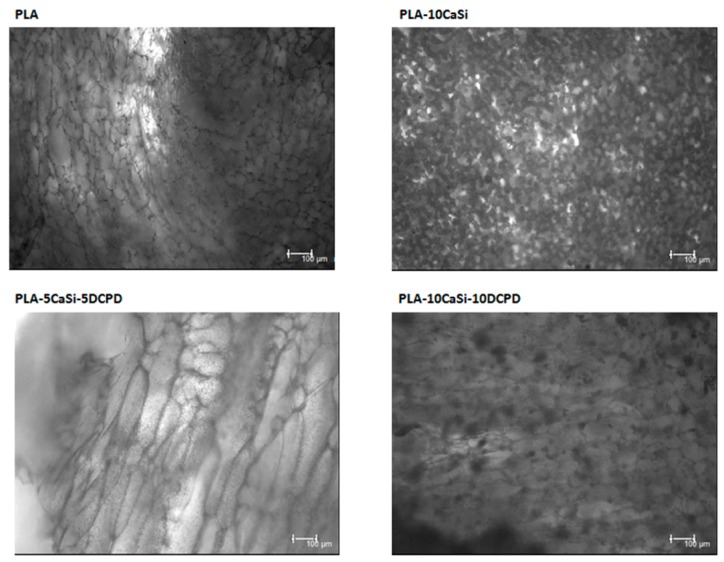
Comparison of different scaffolds microstructure in Bright Field (CLSM, 10× magnification).

As evident in Figure 9, Figure 10 and Figure 11, a good colonization of the scaffolds has been observed in all samples; but a higher number of non-viable cells (red-stained) is clearly observable in PLA-control scaffold at day 14 and at day 21. About hPCy-MSCs seeded on mineral doped scaffolds, PLA-10CaSi, PLA-5CaSi-5DCPD, and PLA-10CaSi-10DCPD, the green stained vital cells predominate at any time-point (day 7–14–21). Figure 12 describes the different microstructures of the analyzed scaffolds observed in Bright Field (B/F), by Confocal Laser Scanning Microscope (CLSM) at 10× magnification.

### 3.5. Osteogenic Differentiation

**Figure 13:**
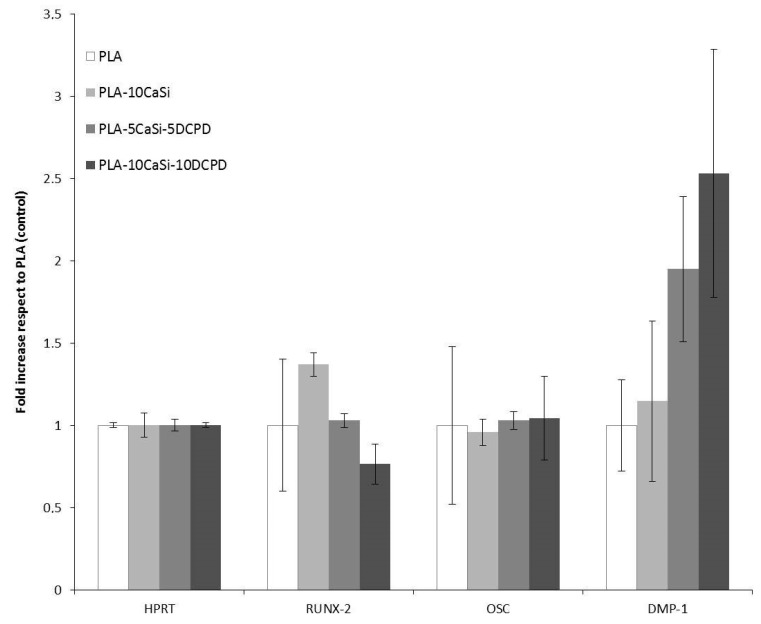
qPCR of hPCy-MSCs after 21 days of culture on mineral doped scaffolds. Histograms indicate fold increase in mRNA levels of hPCy-MSCs cells grown on PLA-10CaSi, PLA-5CaSi-5DCPD and PLA30 10CaSi-10DCPD, with respect to PLA control. Error bars indicate standard deviation of mean values.

In order to test the effects of different novel scaffolds made in our labs, we cultured the hPCy-MSCs on each of them, and we observed the variation of the genes expression closely related to osteogenesis and odontogenesis, by means of quantitative polymerase-chain-reaction. As reported in Figure 13, the expression of osteocalcin, a typical osteogenic marker, is homogeneous in cells seeded in the different experimental scaffolds. The mRNA levels of the early osteogenic marker RUNX-2 are increased in cells grown on PLA-10CaSi scaffold, and they are decreased in cells seeded on PLA-10CaSi-10DCPD. On the other hand, the expression of DMP-1, a well-known odontogenic and osteogenic marker, seems to be increased in hPCy-MSCs cultured on all mineral-doped scaffolds: interestingly, DMP-1 levels are particularly increased in PLA-5CaSi-5DCPD and in PLA-10CaSi-10DCPD scaffolds, revealing the suitability of these two scaffolds in bone regeneration procedures. 

## 4. Discussion

In the present study, hPCy-MSCs were isolated from periapical inflamed cystic tissues and seeded on bioactive mineral-doped novel scaffolds. It has been reported that hPCy-MSCs have key abilities, useful for bone and dental regeneration strategies: the association among these cells and specific osteogenic/odontogenic scaffolds is likely to trigger MSC differentiation towards specific lineages [3]. Clinical applications of such promising association of novel MSCs and novel scaffolds are particularly targeted on regenerative dentistry and biological-guided oral surgery. 

TIPS allowed to produce highly porous composite structures, made of PLA filled with different amounts of CaSi and DCPD, up to 20 wt %. This technique did not induce any modification in the morphological structure of the crystalline fillers upon mixing with the polymer phase, as confirmed by an XRD analysis previously reported in literature [34]. 

TIPS allows to produce extremely highly porous structure with extensive pore interconnectivity [38] and to fabricate scaffolds with a great variation of micro and macropores, although it was difficult to produce pores over 200 µm [39] (structure with pore size 10–100 µm cells proliferation and migration, while 100–1000 µm allow bone ingrowth [40]). In the present study, the high standard deviation (over 30%) (Figure 6) of the wall thickness of the scaffolds, demonstrated a broad range of pore sizes, as reported in other studies on scaffolds production [34,40]. However, trabecular bone also shows a variability of trabecular thickness of approx. 30% [41,42].

PLA drawbacks (hydrophobicity, release of acid degradation products, and reduced cell adhesion and growth) are well-known in literature [18]: the addition of CaSi and DCPD mineral filler significantly improved the biointeractivity, biocompatibility, and apatite forming ability of the materials [34]. Indeed, hPCy-MSCs seeded on the mineral doped scaffolds showed a better colonization, growth, and proliferation on the mineral doped formulations when compared to pure PLA. This behavior is well evident in Figure 9, Figure 10 and Figure 11, where a higher number of dead (red cells) may be observed on PLA scaffolds, in particular at 14 and 21 days.

In our study, a complex micro-CT characterization has been performed: scaffolds pores, their interconnectivity and size, the structural wall thickness, scaffolds anisotropy, and cross-sectional areas had been analyzed, showing a scaffold structure characterized by suitable interconnected pores, where cells were able to migrate, grow, and differentiate. Micro-CT analysis revealed no artefacts or empty areas in the middle part of the mineral-doped scaffold: the presence of mineral fillers was able to reinforce the polymer matrix. This behavior is also confirmed by previous mechanical tests, where pure PLA showed lower values of the elastic modulus, and PLA-10CaSi-10DCPD showed the highest values [34].

The highly porous structure is a key feature for the proper colonization of the mineral-doped scaffold by the cells. Importantly, the mineral compounds used to build the scaffolds were homogeneously distributed: this aspect is important to have a homogeneous bioactivation of the cells along the whole structure of the scaffold.

The evaluation of gene expression, after a long-term culture of oral-derived MSCs with the mineral-doped scaffolds, indicated that an increasing mineralization of scaffolds is closely correlated with the improved expression of the odontogenic/osteogenic marker DMP-1; strategically, we observed the levels of DMP-1 in each scaffold tested, but we would suggest to consider the PLA-10CaSi-10DCPD-based scaffolds as the gold standard, pushing the use of such scaffolds, added with autologous dental-derived MSCs, specifically in bone tissues and dental tissues repairing.

## 5. Conclusions

The most exciting challenge in regenerative dentistry is to find a scaffold able to ensure regenerative healing of periapical and alveolar bone, allowing tooth preservation. In this study, an innovative regenerative clinical approach was conceived through the combination of highly porous, biologically active (biointeractive) scaffolds with autologous hPCy-MSCs. These cells showed a good growth rate and an interesting expression of the typical osteogenic/odontogenic marker DMP-1 on all the mineral-doped scaffolds compared to pure PLA scaffold.

In conclusion, the combination of a bioactive scaffolds colonized with autologous stem cells from periapical cysts may represent a promising strategy for regenerative healing of periapical and alveolar bone.
